# Altered Theta Oscillations and Aberrant Cortical Excitatory Activity in the 5XFAD Model of Alzheimer's Disease

**DOI:** 10.1155/2015/781731

**Published:** 2015-04-02

**Authors:** Magdalena Elisabeth Siwek, Ralf Müller, Christina Henseler, Astrid Trog, Andreas Lundt, Carola Wormuth, Karl Broich, Dan Ehninger, Marco Weiergräber, Anna Papazoglou

**Affiliations:** ^1^Federal Institute for Drugs and Medical Devices (BfArM), Kurt-Georg-Kiesinger-Allee 3, 53175 Bonn, Germany; ^2^Department of Psychiatry and Psychotherapy, University of Cologne, Faculty of Medicine, Kerpener Street 62, 50937 Cologne, Germany; ^3^Institute of Molecular Psychiatry, University of Bonn, Sigmund-Freud Street 25, 53125 Bonn, Germany; ^4^German Center for Neurodegenerative Diseases (DZNE), Ludwig-Erhard-Allee 2, 53175 Bonn, Germany

## Abstract

Alzheimer's disease (AD) is an age-related neurodegenerative disorder characterized by impairment of memory function. The 5XFAD mouse model was analyzed and compared with wild-type (WT) controls for aberrant cortical excitability and hippocampal theta oscillations by using simultaneous video-electroencephalogram (EEG) monitoring. Seizure staging revealed that 5XFAD mice exhibited cortical hyperexcitability whereas controls did not. In addition, 5XFAD mice displayed a significant increase in hippocampal theta activity from the light to dark phase during nonmotor activity. We also observed a reduction in mean theta frequency in 5XFAD mice compared to controls that was again most prominent during nonmotor activity. Transcriptome analysis of hippocampal probes and subsequent qPCR validation revealed an upregulation of Plcd4 that might be indicative of enhanced muscarinic signalling. Our results suggest that 5XFAD mice exhibit altered cortical excitability, hippocampal dysrhythmicity, and potential changes in muscarinic signaling.

## 1. Introduction

Alzheimer's disease (AD) is an irreversible, progressive brain disorder slowly destroying learning and memory skills. The histopathology of AD is characterized by two hallmark lesions, extracellular amyloid-*β* (A*β*) plaques, and intracellular neurofibrillary tangles (NFTs) composed of hyperphosphorylated tau protein. In addition to the presence of A*β* plaques and NFTs in the brain, considerable neuron loss is also a prominent feature of AD, the mechanisms of which still remain unclear. Importantly, familial AD (FAD) mutations in genes for amyloid-*β* precursor protein (A*β*PP), presenilin 1 (PSEN1), and presenilin 2 (PSEN2) implicate A*β* as an initiating factor in AD pathogenesis. These FAD mutations increase the release of A*β*42 from A*β*PP, the latter being sequentially cleaved by the *β*- and *γ*-secretase enzymes to release the A*β*. The latter plays a central role in the pathophysiology of AD and ultimately leads to neuronal cell loss, for example, in the septum, entorhinal cortex, and hippocampus [1, 2], and finally to dementia [3, 4].

Transgenic mouse models have proven to be valuable tools in investigating the etiopathogenesis of AD [5–11]. Studies on various AD mouse models revealed dysregulation of the *β*-site APP cleaving enzyme 1 (BACE1) that is responsible for the generation of A*β* plaques [12–14]. Therefore, increased translation of BACE1 leads to enhanced plaque formation and finally to a disruption of neuronal functioning within the hippocampus [15, 16]. Among different AD models, the 5XFAD model is a most progressive and growth retarded model expressing multiple FAD mutations that additively increase A*β*42 production, that is, three human APP mutations (Swedish mutations, K670N, M671L; Florida mutation, I716V; London mutation, V717I) and two mutant PSEN1 (M146L, L286V). Individually, each FAD mutation enhances A*β*42 generation, but together they act synergistically to predominantly accumulate A*β*42. Thus, 5XFAD mice already exhibit intraneuronal A*β* accumulation at 1.5 months, A*β* deposition at 2 months, and memory deficits at 4 months of age [15, 17–20]. Furthermore, the 5XFAD model is one of a few known mouse models that exhibit significant neuronal loss in the hippocampus that correlates with accumulation of A*β* plaques [15, 21, 22]. However, in the cortex of 12-month-old 5XFAD mice, neuronal cell loss was reported to be predominately related to layer 5 [15, 23]; the overall number of neurons in the frontal cortex and hippocampal CA1 region remained unchanged compared with age-matched wild-type (WT) mice [21]. Thus, cortical plasticity is likely to be impaired prior to hippocampal-dependent learning and memory deficits in 5XFAD mice [24]. It is noteworthy that 12-month-old 5XFAD mice were also reported to exhibit less anxiety, but normal locomotor behavior [21].

Intense efforts were carried out to characterize the transcription and expression profile in 5XFAD mice compared to WT. Using quantitative mass spectrometry to investigate proteome-wide changes in 4-month-old 5XFAD mice [24], alterations were predominantly identified in ApoE, ApoJ (clusterin), and nicastrin expression. NRF2 and p53 transcriptional pathways were activated, as well as IGF-1 signaling. Furthermore, various neurological glial marker proteins and factors implicated in neurological disorders such as AD, Parkinson's disease, Huntington's disease, and amyotrophic lateral sclerosis were affected [25]. Transcriptome analysis has also been carried out for the frontal cortex and cerebellum of 7-week-old 5XFAD mice [26].

Although neuropathological changes in AD have been well described previously, direct effects on systemic electrophysiological alterations have received less attention in the past. Currently, the impact of brain oscillation analysis as a novel tool in early diagnosis and prediction of disease progression is strongly discussed as functional impairments in AD can occur even without any significant neuronal loss and therefore could be independent of plaque formation [27–31]. Recent studies illustrated that altered hippocampal oscillatory activity correlates with an increase of A*β* level and the appearance of plaques [1, 32, 33], but slight changes in hippocampal and cortical network activity can also occur much earlier prior to clinical onset of AD [29–31, 34, 35]. Alterations in network activities in AD are accompanied by an early imbalance of excitation and inhibition that elites overall changes in theta activity as a hallmark of hippocampal functioning [1, 20, 36]. These alterations due to A*β*-induced neuronal hyperexcitability [35] are accompanied by decreased GABAergic transmission within the hippocampus and can trigger seizure activity [37–44]. Neurons being early affected in AD pathogenesis are those of the septohippocampal circuitry, including cholinergic, GABAergic, and glutamatergic cells [45–53]. Interestingly, various mouse models of AD exhibit antithetic alterations in theta rhythmicity; that is, a number of them were reported to present cognitive decline associated with a paradoxical increase in theta activity [27, 54–57] whereas others displayed reduced theta rhythm [55]. The reason for either enhanced or impaired theta activity remains largely unknown. Though recent studies have gained substantial insight into the etiopathogenesis of AD, we are still lacking detailed information about how septohippocampal dysfunction results in altered theta rhythmicity and neuronal hyperexcitability activity in AD.

In this study we combined seizure analysis, time-frequency-based theta analysis, and transcriptome data to elicit systemic cortical and hippocampal network alterations in the 5XFAD model of AD.

## 2. Material and Methods

### 2.1. Study Animals

This study was performed in Tg(APPSwFlLon, PSEN1∗M146L∗L286V) 6799Vas (5XFAD) transgenic mice with a B6/SJL background overexpressing mutant forms of human APP (the Swedish mutations: K670N, M671L; the Florida mutation: I716V; the London mutation: V717I) and mutant* PSEN1* (M146L, L286V). The 5XFAD mice were obtained from The Jackson Laboratory (JAX Mice Strains, USA) [15]. Five WT controls (body weight: 35.32 ± 2.40 g, age: 66.71 ± 7.88 weeks, 4 ♂, 1 ♀) and five 5XFAD mice (body weight: 24.89 ± 1.40 g, age: 72.20 ± 2.77 weeks, all ♂) were analyzed in this study. All mice were housed in groups of 3-4 in clear Makrolon cages type II with ad libitum access to drinking water and standard food pellets. Using ventilated cabinets (Model 9AV125P, Tecniplast, Germany), mice were maintained at a temperature of 21 ± 2°C, 50–60% relative humidity, and on a conventional 12 h light/dark cycle with the light cycle beginning at 5:00 a.m. for spontaneous epidural and deep, intracerebral EEG recordings.

All animal procedures were performed according to the Guidelines of the German Council on Animal Care and all protocols were approved by the Local Institutional and National Committee on Animal Care (Landesamt für Natur, Umwelt und Verbraucherschutz, LANUV, Germany). The authors further certify that all animal experimentation was carried out in accordance with the European Communities Council Directive of November 24, 1986 (86/609/EEC). Specific effort was made to minimize the number of animals used and their suffering.

### 2.2. Stereotaxic EEG Electrode Implantation and Radiotelemetric EEG Recordings

Mice were anesthetized using ketamine/xylazine (100/10 mg/kg i.p.) and the radiotelemetry transmitter (TL11 M2-F20-EET 2-channel transmitter, Data Science International (DSI); specifications: weight 3.9 g, volume 1.9 cc, input voltage range ± 1.25 mV, and channel bandwidth 1–50 Hz) was implanted into a subcutaneous pouch on the back of the animals. The EEG electrodes of both transmitter channels were stereotaxically positioned using a computerized 3D stereotaxic StereoDrive (Neurostar, Germany). The differential epidural electrode of channel 1 targeting the primary motor cortex (M1) was positioned at the following coordinates referring to the bregma craniometric landmark: (+)-lead, cranial +1 mm, and lateral of bregma 1.5 mm (left hemisphere). For deep, intracerebral brain recordings targeting the hippocampal CA1 region the differential electrode of channel 2 was positioned as follows: (+)-lead, caudal −2 mm, lateral of bregma 1.5 mm (right hemisphere), and dorsoventral (depth) 1.5 mm. For both channels, epidural reference electrodes were placed on the cerebellar cortex at bregma −6 mm, lateral of bregma 1 mm (left hemisphere), and bregma −6 mm, lateral of bregma 1 mm (right hemisphere), respectively (see Supplementary Figure 1 of the Supplementary Material available online at http://dx.doi.org/10.1155/2015/781731). Electrodes were fixed using glass ionomer cement (Kent Express, UK) and the scalp was closed using Over and Over sutures (Ethilon, 6–0). To avoid hypothermia, supplemental warmth was given to the animal during the whole surgical procedure. A detailed description of the implantation procedure is given in [58]. For postoperative pain management animals were administered carprofen (5 mg/kg sc., Rimadyl, Parke-Davis/Pfizer, Germany). Animals were allowed to recover for 10 days prior to subsequent recordings. This recovery period is based on the observation that 10 days after surgery no difference in physiological parameters between transmitter implanted, nonimplanted, and sham-operated animals could be detected [59].

### 2.3. Validation of EEG Electrode Placement

Seven controls and six 5XFAD mice were originally implanted. To verify the correct electrode placement targeting the CA1 region, brains were extirpated postmortem and fixed in 4% paraformaldehyde. Afterwards, brains were cut to 60 *μ*m slices using a Vibroslice Tissue Cutter EMS 5000-MZ (Campden Instruments Limited) and hematoxylin-stained for visualization of the branch canal. Two controls and one 5XFAD mouse did not meet strict EEG electrode placement criteria and were thus excluded from the analysis.

### 2.4. EEG Data Acquisition

Ten days after radiotransmitter implantation, simultaneous video-EEG recordings from the motor cortex (M1) and the hippocampal CA1 region were performed for 48 h in all animals from both study groups using Dataquest ART 4.2 software (DSI) at a sampling rate of 500 Hz with no a priori filter cut-off.

### 2.5. Electroencephalographic and Behavioral Seizure Analysis

For further analysis data were exported to NeuroScore 2.1 (DSI). Qualitative and quantitative seizure analysis was performed using the NeuroScore seizure detection module. Spike parameters including dynamic and absolute threshold, spike duration, and spike intervals were adapted for different seizure protocols. Dynamic thresholds were based on multiplication of the root mean square (RMS) values of the EEG signals one minute prior to EEG segments being analyzed. The threshold ratio (minimum threshold) was defined as 2 while the maximum ratio was defined as 15. The minimum amplitude value of spikes was 100 *μ*V for the dynamic range. In case of the absolute threshold the maximum amplitude was fixed at 1000 *μ*V with a threshold value of 200 *μ*V. In both seizure protocols the minimum spike duration was determined at 1 ms and the maximum spike and short/slow-wave duration at 100 ms. Spike trains were detected with a minimum train duration of 0.5 s including at least 4 individual spikes. Spike intervals within a train were ranging between 0.05 and 0.3 s. The interval between individual spike trains was determined at 1 s. Seizure protocols contained the total number of seizure episodes and spikes, spike frequency, total spike train duration, shortest and longest spike train durations. Data were calculated and plotted throughout the figures as mean ± standard error of the mean (SEM). Significance was calculated using Student's *t*-test which included pretesting for normal distributions via the Kolmogorov-Smirnov test.

### 2.6. Urethane Induced Theta Oscillations

Urethane (Sigma, Germany) was freshly dissolved in 0.9% NaCl and systemically (i.p.) administered at 800 mg/kg to induce atropine-sensitive type II theta oscillations. Four control mice from the original study group (body weight: 32.98 ± 0.65 g, age: 70.32 ± 9.04 weeks, 3 ♂, 1 ♀) and four 5XFAD animals from the original study group (body weight: 25.52 ± 1.61 g, age: 73.75 ± 2.97 weeks, all ♂) were used for this approach. CA1 recordings under baseline conditions (30 min duration) and under urethane (30 min duration 15 to 45 min after injection) were used for analysis of urethane induced hippocampal theta oscillations.

### 2.7. EEG Data Analysis

Complex EEG analysis was performed for spontaneous 48 h recordings at a sampling rate of 500 Hz. Data segments with a length of 60 min each were extracted from the 48 h total recordings. Data segments were analyzed using complex Morlet wavelets to calculate both frequency and amplitude of oscillations. The complex Morlet wavelet is defined as

(1) $$\begin{array}{c} \Psi \left(x\right)={\left(\pi b\right)}^{\left(-1/2\right)}\exp\left(2\pi icx\right)\exp\left(-\frac{x^{2}}{b}\right), \end{array}$$

where *b* is the bandwidth parameter, *c* the center frequency, and *i* the imaginary unit [60]. This wavelet or similar ones have often been applied in the literature to study EEG data, as they guarantee optimal resolution in both frequency and time [60, 61]. In our case, the bandwidth parameter and center frequency were both set to 3 in order to weight the frequency resolution to distinguish frequency differences on the 0.1 Hz level, but not to neglect a sufficient time resolution. EEG data were analyzed in the frequency range of 0.2–12 Hz with a step size of 0.1 Hz, thus including the typical delta, theta, and alpha frequency ranges. In order to apply the wavelet technique for extraction of theta-oscillatory segments, we developed a task-adjusted detection criterion. This theta detection method mimicked the standard visual inspection of theta oscillations and was substantially based on a complex elaboration of the frequency architecture of theta activity [62].

Theta-positive oscillatory segments of 2.5 s duration were defined as follows (*A*_max⁡_: maximum amplitude):

(2) $$\begin{array}{c} \frac{A_{\text{max(4–12 Hz)}}\left(\text{mV}\right)}{A_{\text{max(2–3.9 Hz)}}\left(\text{mV}\right)}>2. \end{array}$$

The 2.5 s time length is suited to precisely analyze theta activity [20]. Some extracted theta segments did not correspond to normal theta activity and were related to spontaneous behavior, for example, eating or scratching as proven by video analysis of the mice. To eliminate these segments, a specific algorithm was developed and validated. All theta segments with a mean frequency lower than 5 Hz or higher than 10 Hz, exhibiting only slight frequency changes over time, were excluded from the analysis. Finally, sorted EEG segments identified as theta oscillation epochs were statistically analyzed and all data were displayed as mean ± SEM. Furthermore, activity data of mice during 48 h recordings and the conventional 12 h light/dark cycle (beginning at 5:00 a.m.) were used to correlate theta activity from the CA1 region with either the active or nonactive state. All EEG calculations were done using custom-made programs in MATLAB (The MathWorks Inc., Version R2012b).

### 2.8. Gene Expression Profiling Using Microarray Procedure

Total RNA (250 ng) was prepared from the hippocampi of three independent control mice (age: 68.10 ± 0.05 weeks, 2 ♂, 1 ♀) and three independent 5XFAD animals (age: 68.24 ± 0.75 weeks, 2 ♂, 1 ♀) that were not involved in any other experimental procedures before using RNeasy Lipid Tissue Mini Kit (Qiagen). Microarray experiments were carried out using Mouse Exon ST arrays (Affymetrix). Arrays were washed and stained according to the manufacturer's recommendations. Labeled and purified cDNA was fragmented (5.5 *μ*g) and subsequently hybridized to the arrays before scanning in a GeneChip 3000 7G scanner (Affymetrix). Normalization to the median of all samples, background correction, and statistical analysis were performed with GeneSpringGX software (Agilent technologies). An implemented GC-RMA algorithm was applied on all chips to summarize probe level information. Microarray data were analyzed using unpaired *t*-tests. An uncorrected significance level *P* < 0.05 was adopted in all instances. Differentially regulated transcripts with a fold change (FC) greater than 1.6 were then subject to hierarchical clustering analysis in order to visualize gene expression changes across groups. Array data are available in the GEO database under GSE50521. DAVID [63, 64] was used to carry out gene ontology enrichment analyses in the gene set differentially expressed between 5XFAD and WT controls.

### 2.9. RNA Extraction and Quantitative Real-Time PCR (qPCR)

Quantitative real-time PCR was used to validate potential gene candidates that exhibited transcriptional alterations in microarray analysis. The cDNA synthesis from hippocampal RNA (see above) was carried out using anchored-oligo(dt)18 and hexamer primer in a two-step RT-PCR approach (Transcriptor First Strand cDNA Synthesis Kit, Qiagen) and qPCR reaction protocol was based on LightCycler 480 SYBR Green I Master (Roche). The qPCR was performed in a Light Cycler 480 System (Roche) thermocycler. The following cycler protocol was used for all primer pairs (Table 1): 95°C (10 min, preincubation step); 95°C (10 s, melting step); 60°C (20 s, annealing step); and 72°C (30 s, extension step), 35 cycles. The specificity of the amplification was checked by melting curve analysis and the products were identified by electrophoresis. Deionized, nuclease-free water (no cDNA) and total RNA samples (without RT) were used as controls and HPRT was used as an internal reference gene. The Ct (cycle threshold) values were calculated using the LightCycler 480 System software. Fold changes (FC) of Cacna2d1, Kcnma1, Cacna1e, Prkcb, Plcd4, Scn8a, Plcb1, and Casp8 gene expression in 5XFAD transgenic mice related to WT controls were calculated according to [65].

### 2.10. Statistical Analysis

Further statistical analyses concerning duration, frequency, amplitude of theta segments, and comparisons between groups and experimental conditions were done with IBM SPSS Statistics, Version 22 (IBM Corporation, 2013). The Kolmogorov-Smirnov test was used to test for normal distributions. Student's *t*-test was used to detect differences between the two groups of the above-mentioned parameters. For data that did not show a normal distribution, the Mann-Whitney *U* test was used instead, where we made use of the exact solution. This is necessary since the asymptotic solution overestimates the *P* value for small numbers of animals per group. For analysis of urethane induced theta oscillations the repeated measures ANOVA with within-subjects factor, that is, experimental condition (baseline versus after urethane) and between-subjects factor, that is, genotype (control mice versus 5XFAD mice) was applied. The Simes-Hochberg “step-up” procedure was used to correct for multiple testing, if necessary [66]. This procedure was utilized at a *P*-level of 0.05.

## 3. Results

### 3.1. Phenotypical Characterization

As reported previously, 5XFAD mice exhibited a reduced body weight in comparison to their WT littermates (24.89 ± 1.40 g, *n* = 5 v. 35.32 ± 2.40 g, *n* = 5, df = 8, *t* = 3.757, and *P* = 0.006). The same holds true for a characteristic clasping phenotype involving a simultaneous retraction of both fore- and hindpaws [21].

### 3.2. Electrocorticographic Characteristics of Control and 5XFAD Mice

**Figure 1:**
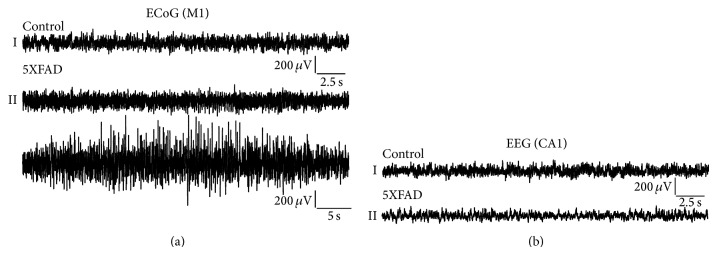
Electroencephalographic (EEG) characteristics of controls and 5XFAD mice. EEGs recorded from the primary motor cortex (M1) as electrocorticogram (ECoG) (a) and the hippocampal CA1 region as electrohippocampogram (b) from both WT and 5XFAD mice. In the CA1 electrohippocampogram neither controls (b)I nor 5XFAD mice (b)II exhibited typical ictal discharges in 48 h long-term recordings. Electrocorticographic M1 recordings, however, exhibited subclinical, that is, electroencephalographic seizure activity in 5XFAD mice of different severity (a)II that was not detectable in control mice (a)I. (a)I, (b)I, and b(II): 30 sec duration; (a)II 50 sec duration.

In this study epidural M1 and deep intrahippocampal CA1 long-term (48 h) EEG recordings were obtained in 5XFAD (*n* = 5) and WT (*n* = 5) mice (Figure 1). EEG recordings were combined with simultaneous video-recordings to detect potential movement artefacts and to differentiate convulsive from nonconvulsive seizure activity. 5XFAD mice exhibited aberrant hyperexcitability in the M1 recording depicting typical episodes or trains of spike, polyspike, and spike-wave activity (Figure 1(a)II) whereas WT mice did not (Figures 1(a)I and 1(b)I). Video analysis revealed that none of the motor cortex seizures were associated with motoric exacerbation, thus remaining subclinical or nonconvulsive.

### 3.3. Seizure Analysis in Control and 5XFAD Mice

**Figure 2:**
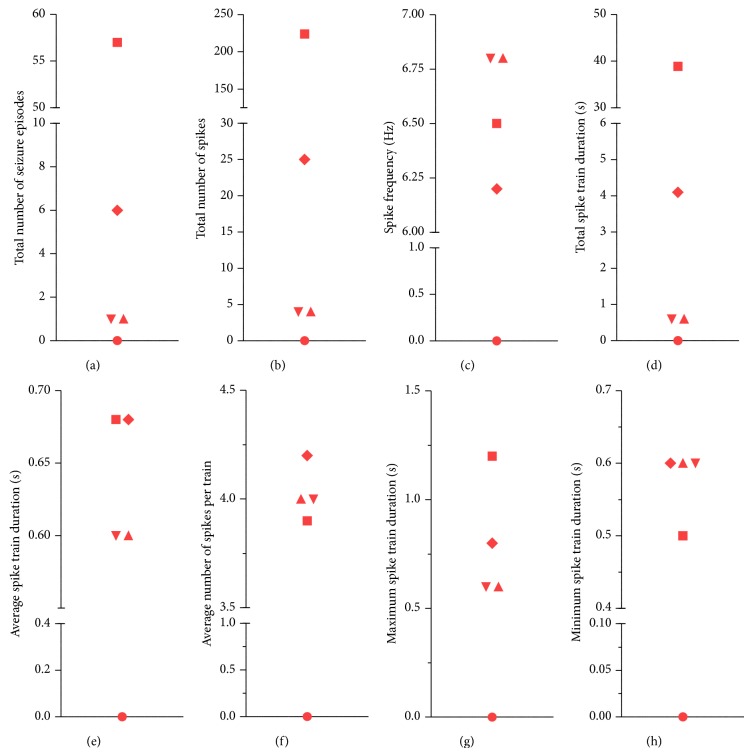
Seizure characteristics in control and 5XFAD mice. Dot plots illustrating results for individual 5XFAD mice. Total number of seizure episodes (a) and spikes (b), spike frequency (c), total (d) and average (e) spike train duration, average number of spikes per train (f), and maximum (g) and minimum spike train duration (h) are depicted. Parameters were analyzed using the NeuroScore Automated Seizure Module (DSI). Note that 5XFAD mice exhibited ictal discharges of highly variable degree whereas none of the controls displayed ictal-like discharges (not shown).

The seizure phenotype of 5XFAD mice (*n* = 5) and WT mice (*n* = 5) was analyzed using simultaneous video-EEG recordings for a total duration of 48 h (Figure 2). None of the WT littermates exhibited aberrant hyperexcitability according to the automated seizure scoring system (NeuroScore 2.1, DSI). In contrast, 5XFAD mice displayed ictal-like discharges, such as spikes, polyspikes, and spike-waves. These discharges were capable of forming seizure episodes characterized by seizure initiation, seizure prolongation, and seizure termination. Interictal discharges originated from single spike activity in most cases. None of this aberrant hyperexcitability was accompanied by apparent motoric exacerbation, for example, forelimb clonus, rearing and falling, or generalized tonic-clonic seizures. However, minor motor activity including, for example, orofacial clonus or partial isolated clonus or tonus of the limbs, can be very subtle and thus cannot be fully excluded. In summary, the total number of seizure episodes was 13.00 ± 11.05, the total number of spikes: 51.40 ± 43.37, the spike frequency: 5.26 ± 1.32 Hz, and the total spike train duration: 8.84 ± 7.55 sec (Figures 2(a)–2(d)). Further seizure parameters in 5XFAD mice included the spike train duration of 0.51 ± 0.13 sec, the number of spikes per train of 3.22 ± 0.81, the maximum spike train duration of 0.64 ± 0.19 sec, and the minimum spike train duration 0.46 ± 0.12 sec (Figures 2(e)–2(h)). These seizures turned out to be subclinic or nonconvulsive and were prominent in the M1 deflection. However, a single 5XFAD mouse exhibited convulsive tonic-clonic seizures of status-like character with prominent interictal spike activity (not shown) and died during the early recovery period. Thus, it was excluded from further analysis. Interestingly, 5XFAD mice did not show ictal discharges in the deep, intrahippocampal CA1 recording (Figure 1(b)I). Disinhibitory tendencies as being shown in our seizure analysis were speculated to be relevant also for reduced anxiety in 5XFAD mice [21].

### 3.4. Differential Gene Expression Detected by Microarray Analysis

Alzheimer's disease is associated with considerable transcriptional alterations in key brain areas. Gene expression analysis employing Mouse Exon ST arrays (Affymetrix) on hippocampal tissue revealed 1421 transcripts differentially regulated between 5XFAD mice and WT controls. The statistical analysis applied an uncorrected significance level of *P* < 0.05. Array data and further detailed information are available in the GEO database under GSE50521. Candidates which are likely to be of interest for seizure and theta activity are depicted in Supplementary Table 1. Those with FC > 2 are listed in Supplementary Table 2. Gene ontology analysis revealed that the strongest transcriptional alterations belong to immune-response and inflammation related genes observed in the context of late-stage cerebral amyloidosis.

### 3.5. Changes in Gene Expression Levels in 5XFAD Mice Compared with WT Controls

**Figure 3:**
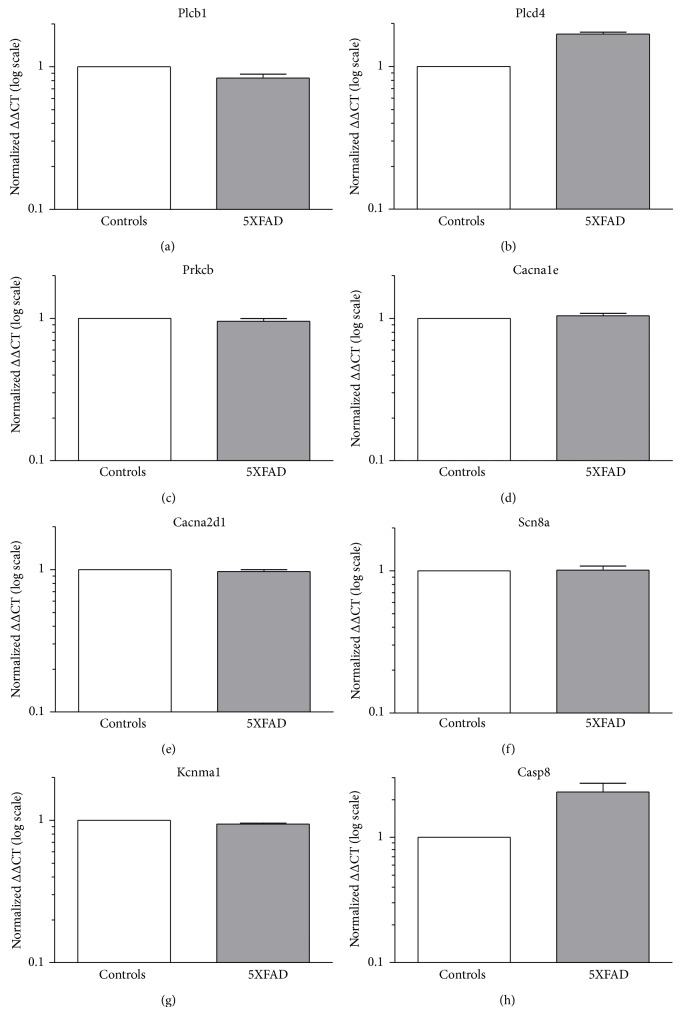
Gene transcription profiles in 5XFAD mice. RNA extracted from the hippocampus of control and 5XFAD mice was used for microarray analysis. A selected number of candidates which exhibited altered expression profile in microarray analysis were further validated using quantitative real-time PCR (qPCR). Normalized ΔΔCT (log scale) for Cacna2d1, Kcnma1, Ca_v_2.3_II-III_-loop, Prkcb, Plcb1, Scn8a, Plcd4, and Casp8 is depicted. Note that a minor decrease in Plcb1 transcript levels turned out to exist, but a clear increase in Plcd4 in 5XFAD mice.

We performed qRT-PCR on hippocampi from three independent WT controls and three independent 5XFAD mice to determine gene expression changes. Among the differentially expressed genes in the microarray assay, we chose eight genes for qRT-PCR analysis based on their involvement in the theta-genesis pathway. The selected genes and their qPCR primers are listed in Table 1. Among the eight genes tested, qPCR revealed upregulation of Casp8 (FC: 2.0979, Table 2) which is likely to be responsible for neuronal cell loss in 5XFAD mice based on altered regulating of microglia activation through a PKC-*δ* dependent pathway [67]. The Scn8a Na^+^ channels as well as Kcnma1 K^+^ channels were not changed in transcription. Interestingly, microarray analysis suggested potential alterations in the muscarinic signal transduction pathway including Plcb1, Plcd4, Prkcb, Cacna1e, and Cacna2d1 that might be relevant for theta oscillations [62]. Validation of these components finally supported an increase in Plcd4 transcript levels (FC: 1.6105) whereas no substantial fold change could be detected for the other factors (Figure 3, Table 2).

### 3.6. Intrinsic Hippocampal Oscillatory Activity in WT Control and 5XFAD Mice

**Figure 4:**
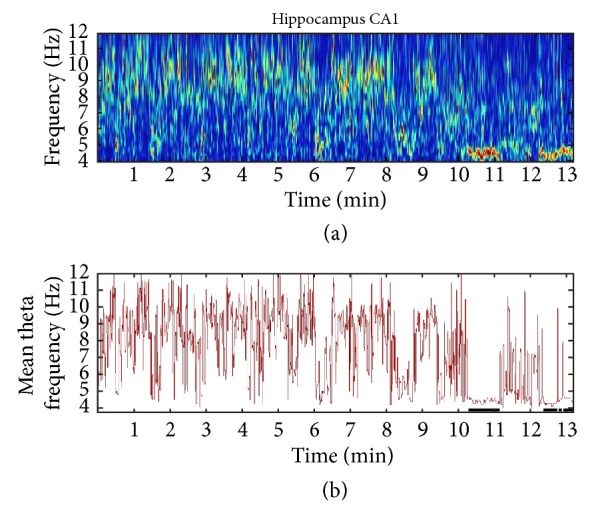
Time-frequency analysis of theta activity in control and 5XFAD mice. (a) Color-coded time-frequency plot of extracted theta segments from EEG data. These segments were clued together (13 minutes in total) for a better demonstration of the oscillatory activity. The *y*-axis represents the frequency range of 4–12 Hz. (b) Mean theta frequency (Hz) calculated for the hippocampal theta-alpha band (4–12 Hz). Note that (a) and (b) display specific episodes of consistent, high amplitude, low frequency EEG activity that was proven to be related to behavioral aspects such as grooming, scratching, or eating based on video analysis. A specific algorithm was defined to automatically detect and eliminate these episodes from further evaluation (black bars, (b)).

**Figure 5:**
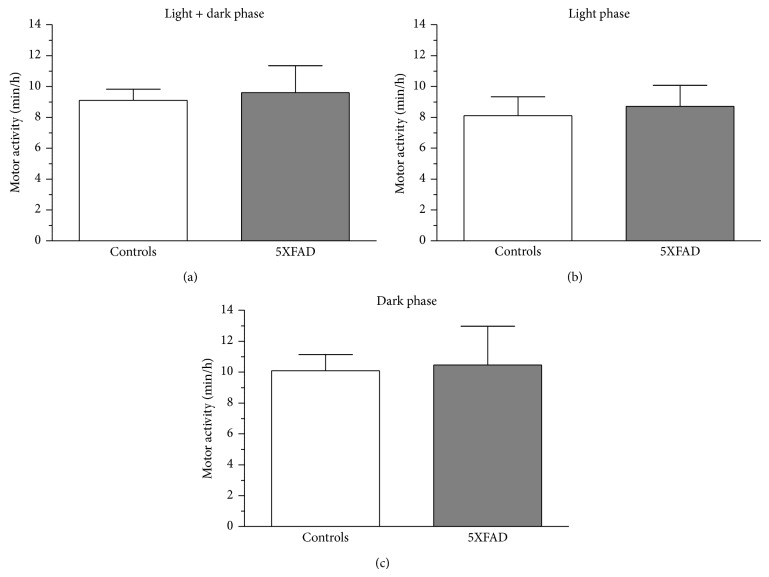
Motor activity in controls and 5XFAD mice. The radiotelemetry system is capable of measuring movement in the horizontal plane as relative units. 10 s epochs were categorized in a binary fashion as motor segments or nonmotor segments. Motor activity was then calculated as (min/h) for the whole observation period (light + dark phase, (a)) and the light (b) and dark phases (c) separately. No difference was observed between both genotypes.

Prior to analysis of the hippocampal oscillatory behavior, we analyzed motor activity in control and 5XFAD mice as active exploratory behavior is associated with a different type of theta entity as compared to, for example, alert immobility (Figure 4). Movement in the horizontal plane was automatically determined by the recording system. The total time of motor activity did not change between controls and 5XFAD mice (9.10 ± 0.72 min/h versus 9.59 ± 1.77 min/h, Figure 5(a)). The same holds true for motor activity during the light phase (8.11 ± 1.23 min/h versus 8.72 ± 1.36 min/h, Figure 5(b)) and dark phase (10.09 ± 1.05 min/h versus 10.46 ± 2.52 min/h, Figure 5(c)). These results correlate with findings from [21] for exploratory and spontaneous locomotor activity in 5XFAD mice aged 9 to 12 months.

As expected, however, there was an increase in motor activity from light to dark phase in both genotypes (Figures 5(b) and 5(c)). Based on these findings, alterations in 5XFAD theta architecture cannot be attributed to changes in activity pattern.

**Figure 6:**
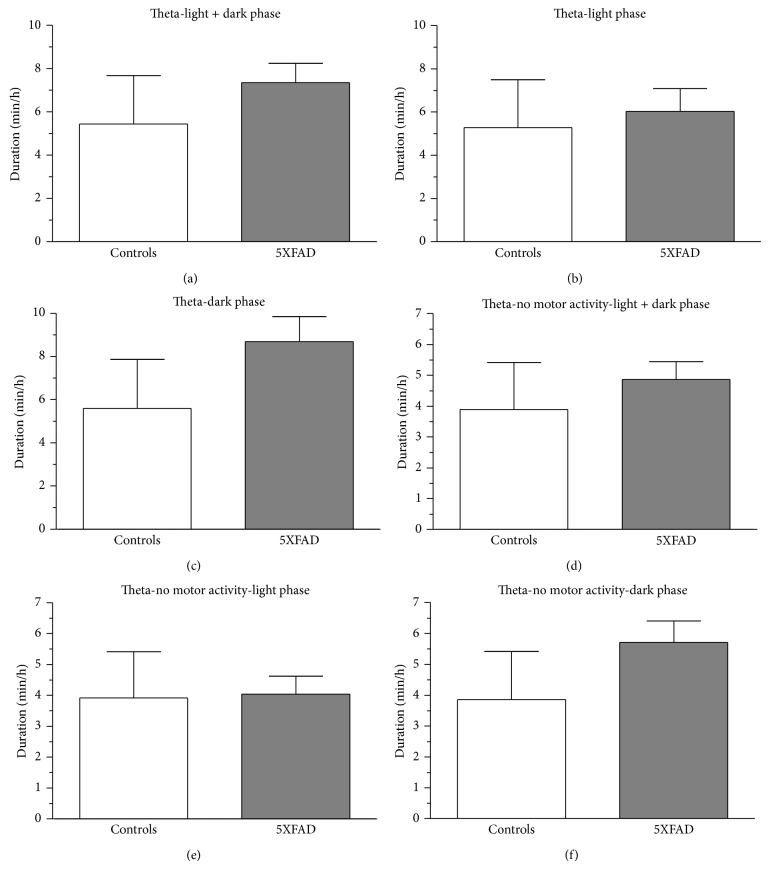
Characteristics of theta duration in control and 5XFAD mice. The theta-alpha band was analyzed using a time-frequency approach. Behavioral artefacts were removed using an algorithm as described above. Theta-segments (10 s) were summed to determine theta duration (min/h). Theta duration was calculated for both genotypes for the total observation period (a) and the light (b) and dark (c) phases, respectively. Total and phase-specific analysis was also done for nonmotor activity ((d)–(f)) and motor activity (not shown). Mean values suggest an increase in theta duration in 5XFAD mice, particularly during the dark phase ((c), (f)).

**Figure 7:**
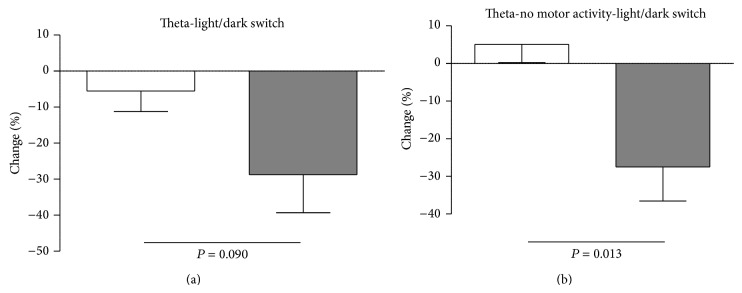
Percentage change in theta duration during light/dark switch. Data from Figures 6(b), 6(c), 6(e), and 6(f) were used to calculate the percentage change in theta duration for every individual mouse during the total observation period (48 h, (a)), during nonmotor activity (b) and motor activity (not shown). During switch from light to dark cycle a statistic trend for theta duration increase turned out to exist in 5XFAD mice (a). Again, this effect was most prominent and significant during no motor activity (b) and absent during motor activity.

To determine whether hippocampal theta oscillations were indeed altered in 5XFAD mice, we performed spontaneous 48 h video-EEG recordings from the CA1 region of the hippocampus from both controls and 5XFAD mice. Using a time-frequency approach, theta duration, theta frequency, and theta amplitude were calculated. First, theta duration was analyzed for both motor and nonmotor activity. No significant changes were observed for controls compared to 5XFAD mice during the total observation period (5.44 ± 2.24 min/h versus 7.36 ± 0.89 min/h, Figure 6(a)), the light phase (5.29 ± 2.21 min/h versus 6.03 ± 1.06 min/h, Figure 6(b)), or the dark phase (5.59 ± 2.27 min/h versus 8.69 ± 1.16 min/h, Figure 6(c)). However, data suggested that there might be an increase in theta during the dark phase (Figure 6(c)). During nonmotor activity again no significant differences were detected for the total duration (3.89 ± 1.53 min/h versus 4.87 ± 0.57 min/h, Figure 6(d)), the light phase (3.91 ± 1.50 min/h versus 4.03 ± 0.59 min/h, Figure 6(e)), or the dark phase (3.86 ± 1.56 min/h versus 5.71 ± 0.70 min/h, Figure 6(f)). During the dark phase of no motor activity a tendency of increased theta duration was observed again, particularly in relation to the light phase. Based on this observation we analyzed the percentage change of theta from light phase (LP) to dark phase (DP) via (LP/DP − 1)∗100. For the total 48 h observation period, a significant trend was observed between controls and 5XFAD mice (−5.55 ± 5.77 versus −28.77 ± 10.56, df = 8, *t* = 1.928, *P* = 0.090, Figure 7(a)); for nonmotor activity this change turned out to be significant (5.11 ± 4.88 versus −27.51 ± 8.99, df = 8, *t* = 3.189, *P* = 0.013, Figure 7(b)). Theta analysis during motor activity did not reveal any statistical differences (light and dark: 1.56 ± 0.72 min/h versus 2.49 ± 0.48 min/h; light: 1.38 ± 0.72 min/h versus 2.00 ± 0.50 min/h; dark: 1.73 ± 0.76 min/h versus 2.98 ± 0.74 min/h).

**Figure 8:**
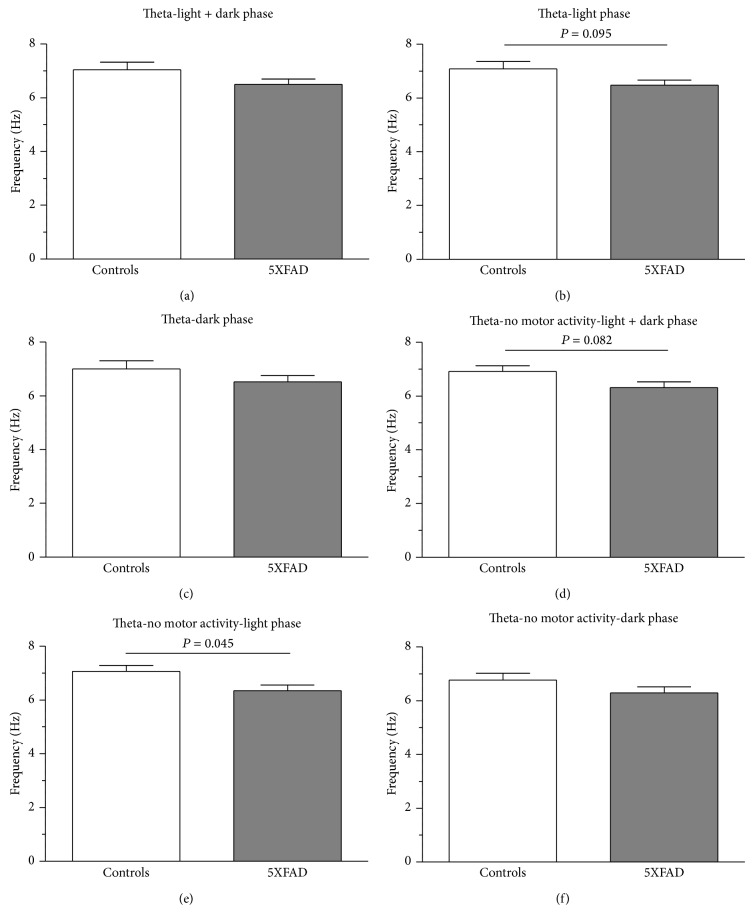
Theta frequency in controls and 5XFAD mice. The mean theta frequency was calculated for the total observation period (48 h, (a)–(c)), no motor activity ((d)–(f)), and motor activity (not shown). In all cases, theta frequency is reduced. Again, this reduction turned out to be significant during no motor activity (e).

An important parameter to be affected during the pathogenesis of Alzheimer's disease in humans is theta frequency [68–71]. For the total recording period (light and dark phase) and the dark phase there was a tendency of reduced theta frequency in 5XFAD mice (7.04 ± 0.28 Hz versus 6.50 ± 0.20 Hz, Figure 8(a); 6.77 ± 0.25 Hz versus 6.29 ± 0.23 Hz, Figure 8(c)) with a statistical trend during the light phase (7.09 ± 0.26 Hz versus 6.48 ± 0.19 Hz, df = 8, *t* = 1.896, *P* = 0.095, Figure 8(b)). For the nonmotor activity, the same phenomenon was observed with no significant change during the dark phase (6.77 ± 0.25 Hz versus 6.29 ± 0.23 Hz, Figure 8(f)), a significant trend for the total observation period (6.92 ± 0.22 Hz versus 6.32 ± 0.21 Hz, df = 8, *t* = 1.989, *P* = 0.082, Figure 8(d)) and a significant reduction during the light phase (7.06 ± 0.22 Hz versus 6.35 ± 0.21 Hz, *P* = 0.045, Figure 8(e)). No significant differences in theta frequency were observed for motor activities (light and dark: 7.17 ± 0.38 Hz versus 6.69 ± 0.18 Hz; light: 7.12 ± 0.33 Hz versus 6.61 ± 0.18 Hz; dark: 7.23 ± 0.42 Hz versus 6.76 ± 0.23 Hz).

Finally, the mean theta amplitudes were calculated. For the total recording period, no changes in theta amplitude could be detected (light and dark: 0.019 ± 0.004 mV versus 0.018 ± 0.003 mV, light: 0.019 ± 0.004 mV versus 0.018 ± 0.003 mV; and dark: 0.019 ± 0.004 mV versus 0.018 ± 0.003 mV, Supplementary Figure 2(A–C)). The same holds true for the nonmotor activity (light and dark: 0.020 ± 0.004 mV versus 0.018 ± 0.003 mV; light: 0.020 ± 0.004 mV versus 0.018 ± 0.003 mV, and dark: 0.019 ± 0.004 mV versus 0.018 ± 0.003 mV, Supplementary Figure 2(D–F)) and nonmotor phase. This finding correlates with previous observations in 5XFAD but also TgCRND8 mice [20].

### 3.7. Urethane Induced Hippocampal Theta Oscillations in Controls and 5XFAD Mice

**Figure 9:**
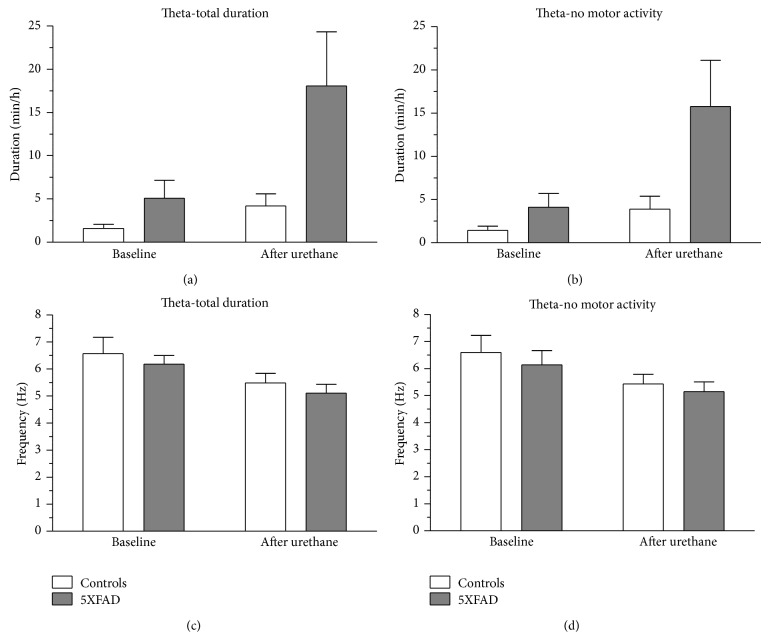
Urethane induced hippocampal theta oscillations in control and 5XFAD mice. CA1 hippocampal theta recordings from both genotypes were analyzed for duration of atropine-sensitive type II theta oscillations regarding total observation period (a) and nonmotor episodes (b). In addition, theta frequency was calculated for total duration (c) and nonmotor episodes (d).

Besides analysis of spontaneous theta activity, we also investigated urethane induced theta oscillations in 5XFAD (*n* = 4) and WT mice (*n* = 4). Pharmacodynamically, urethane has a multitarget character capable of inducing atropine-sensitive type II theta. Theta oscillations were analyzed for 30 min baseline and 30 min postinjection episodes (Figure 9). The duration of hippocampal theta oscillations for the total analytical period, that is, including theta oscillations during episodes with either motor or nonmotor activity, was increased in control mice (1.59 ± 0.47 min/h to 4.20 ± 1.40 min/h, *n* = 4) as in 5XFAD mice (5.09 ± 2.06 min/h to 18.08 ± 6.25 min/h, *n* = 4) and the factor “experimental condition/urethane effect” exhibited significance (*F*(1,6) = 7.270, *P* = 0.036). The “genotype” factor exhibited a statistical trend (*F*(1,6) = 5.256, *P* = 0.062, Figure 9(a)). As type II theta is characteristic of alert immobility data were also analyzed for nonmotor activity EEG segments. As expected, the results matched those for the total analytical period, that is, a significant effect for the factor “experimental condition/urethane effect” (*F*(1,6) = 7.691, *P* = 0.032) with increase in controls (1.45 ± 0.48 min/h to 3.88 ± 1.52 min/h, *n* = 4) and 5XFAD mice (4.11 ± 1.59 min/h to 15.78 ± 5.34 min/h, *n* = 4). The factor “genotype” exhibited a statistical trend (*F*(1,6) = 5.148, *P* = 0.064, Figure 9(b)). These findings demonstrate that 5XFAD mice even of higher age are capable of displaying increased theta oscillations upon urethane provocation. However, it remains to be determined whether such theta activity is physiologically integrated or the result of hyperactive, functionally dislinked neuronal clusters within the hippocampus. Interestingly, besides an effect on theta duration, there was a significant effect for the factor “experimental condition/urethane effect” (*F*(1,6) = 13.536, *P* = 0.010) on theta oscillation frequency for the total analytical period (6.57 ± 0.61 Hz to 5.48 ± 0.36 Hz (*n* = 4) in controls versus 6.18 ± 0.31 Hz to 5.11 ± 0.33 Hz (*n* = 4) in 5XFAD mice, Figure 9(c)). This significant effect (*F*(1,6) = 8.036, *P* = 0.030) also holds true for the nonmotor episodes (6.60 ± 0.63 Hz to 5.44 ± 0.35 Hz (*n* = 4) in controls versus 6.14 ± 0.53 Hz to 5.15 ± 0.36 (*n* = 4) in 5XFAD mice (Figure 9(d)).

## 4. Discussion

Alzheimer's disease is a complex neurodegenerative disorder accompanied by cognitive impairment that ultimately leads to dementia. In the present study, we investigated transcriptional alterations and the EEG phenotype of 5XFAD mice. This mouse model harbors five early-onset FAD mutations and displays substantial amyloid plaques and neurodegeneration [17, 23]. Our study demonstrates that 5XFAD mice exhibit nonconvulsive seizure activity of highly different severity, predominantly in the M1 deflection, whereas there was no seizure activity detectable in the CA1 recordings. It is noteworthy that A*β* formation in certain mouse models can differentially alter cholinergically induced rhythmicity according to the structure of A*β* plaques [17] and therefore resulting in variable seizure severity. In addition, stereological quantification of pyramidal neurons of the CA1 layer showed no significant difference between the number of neurons of WT and 5XFAD mice; however a significant loss was detected in cortical layer 5 [17]. Experimental studies in genetically engineered mice support these findings, highlighting the presence of subclinical seizures and overlapping pathophysiological cascades [72]. Reduced seizure thresholds and spontaneous convulsive seizure phenotypes have been observed in AD mouse models [73–76]. Interestingly, deletion of A*β*PP [77] and BACE1, the secretase that participates in A*β* release [78], also causes an epileptic phenotype, indicating that normal A*β*PP signaling is important for the development of hippocampal excitability [35]. In some models, lower thresholds for induced or spontaneous seizures are found even in the absence of amyloid deposits, further stressing the role of soluble forms of A*β* as a pathogen [76, 77]. Chronic EEG monitoring in J20 mice overexpressing hA*β*PP [31] revealed that most frequent seizures were purely electroencephalographic, that is, nonconvulsive with complete motoric arrest as observed in our study using 5XFAD mice [30, 31]. In rare cases motor seizures were observed. This observation raises the question whether abnormal hippocampal neuronal synchronization might remain undetected in human AD patients and whether altered network activity might accelerate a more rapid cognitive decline in patients suffering from FAD [29, 31, 79]. Histological evaluation of the J20 mouse hippocampus revealed evidence for hippocampal network remodeling that is similar, but not identical, to the changes identified in both patients with temporal lobe epilepsy and experimental models of hippocampal seizures. The cellular changes included ectopic sprouting of dentate granule cell mossy fibres and sprouting of fibers containing the inhibitory neurotransmitter NPY [80, 81]. Convulsive seizures with associated hippocampal network plasticity have been confirmed in other AD mouse models [35, 82]. Interestingly, impairment of GABA transmission in the J20 brain is likely to be responsible for epileptogenesis in these models. Increased adult neurogenesis is found in both human AD and TLE cases [81, 83]. The multiple lines of evidence discussed above reveal that soluble forms of A*β* are cytotoxic inducing the appearance of aberrant excitatory neuronal network activity* in vivo* and triggering complex molecular and cellular patterns of compensatory inhibitory and excitatory mechanisms in the hippocampal circuitry [20, 43, 79, 84, 85].

The toxic accumulation of A*β* peptides underlying AD triggers synaptic degeneration, circuit remodeling, and abnormal synchronization within the same networks. Because neuronal hyperexcitability amplifies the synaptic release of A*β*, seizures create a vicious spiral that accelerates cell death and cognitive decline in the AD brain [34]. While degenerative processes in the nervous system ultimately result in loss of neural signaling, when active inhibitory mechanisms fail early, the resulting disinhibition may destabilize network oscillatory activity at formative stages of the disease.

How can seizure activity correlate with altered theta oscillations in the hippocampus? Complex cognitive operations depend on a sophisticated coordination of activity across a plethora of neuronal groups. One of the most intriguing mechanisms of neuronal coordination and communication is through neuronal synchronization by brain oscillations [86]. Theta oscillations represent one of these oscillations and are modulated by specific behavioral and cognitive states and are related to memory deficits, for example, in AD [31, 32, 34, 49, 56]. Disruption of theta activity results in spatial memory deficits, whereas the restoration of theta-like rhythmicity restores learning capabilities in rats [87]. Theta duration analysis in our study suggests an increase of theta oscillations in 5XFAD mice during the dark phase. This phenomenon was most prominent during the switch from light to dark phase and nonmotor activity. As analysis of motor/nonmotor activity did not reveal any difference between controls and 5XFAD mice; the difference in theta duration is likely to be based on theta-subtype composition. We speculate that nonactive mice in the dark phase predominately exhibit alert-immobility which is accompanied by atropine-sensitive type II theta [88, 89]. As in our study, a statistical trend for an increase in theta duration has been described in the TgCRND8 mouse model [20]. These changes in theta duration in 5XFAD mice are an important variable because they are known to be associated with memory effectiveness. Animals displaying a higher amount of theta activity are faster in novel task learning than animals that exhibit less pronounced theta oscillations [90]. Unlike the common assumption of decreased theta activity in dementia, an increase in theta activity is observed in various mouse models of AD. Administration of urethane clearly proved that 5XFAD mice can exhibit increased atropine-sensitive type II theta oscillations upon provocation (Figures 9(a) and 9(b)). One might speculate that hyperexcitable neuronal clusters surrounded by degenerated neurons might account for this increase in theta which disrupts the ability of neuronal networks to dynamically adjust the amplitude of these oscillations during memory processes. Interestingly, the mean theta frequency was reduced in 5XFAD mice; that is, theta oscillations turned out to be significantly slower than in control animals. This phenomenon is commonly observed in AD patients [68–71]. It has further been suggested that theta frequency could change as a function of novelty and familiarity [91]. Thus, the significant decrease in theta frequency could dramatically skew the delicate balance of theta frequency dynamics between novelty and familiarity.

Previous studies have shown that the muscarinic signal transduction cascade [92] is severely affected in AD [93] and this might correlate with a very slight downregulation of Plcb1 as observed in our study (Supplementary Figure 3). Ca_v_2.3 voltage-gated Ca^2+^ channels are involved in both the generation of cellular correlates of ictal discharges, that is, after depolarisation and plateau potentials [94, 95], and the generation of atropine-sensitive type II theta [62, 88, 96, 97]. Transcriptome analysis of 5XFAD hippocampal probes performed in this study suggests an upregulation of Plcd4 which could result in type II theta acceleration via the PKC, Ca_v_2.3 cascade [62]; however, other muscarinic signaling targets could also be involved (Supplementary Figure 3).

Our study demonstrates that 5XFAD mice exhibit both nonconvulsive seizure activity and altered theta architecture, the latter being related to atropine-sensitive type II theta. Based on hippocampal microarray analysis we hypothesize that altered muscarinic signaling might play a role in the pathophysiology of both alteration. Although some caution is warranted in interpreting results from the aggressive 5XFAD model, our results support the view that pharmacological interference with muscarinic signaling is a potential valuable target in AD treatment. Finally, our study suggests that alterations in theta characteristics might serve as a diagnostic and prognostic biomarker in AD in the future.

## Supplementary Material

Methodological aspects of stereotaxic EEG electrode placement, analysis of theta amplitude, and microarray analysis in WT and 5XFAD mice.

## Figures and Tables

**Table 1:** Sequence of primer pairs used for qPCR.

Gene	Forward sequence	Reverse sequence	Accession number	Size (bp)
HPRT^1^	GCTGGTGAAAAGGACCTCT	CACAGGACTAGAACACCTGC	J00423	249
Kcnma1^2^	CCTGAAGGACTTTCTGCACAAGG	ACTCCACCTGAGTGAAATGCCG	NM_010610	122
Cacna2d1^2^	GTGGAAGTGTGAGCGGATTGAC	TCGCTTGAACCAGGTGCTGGAA	NM_001110843	150
Prkcb^2^	CCAAGATGACGATGTGGAGTGC	CTCCATCACAAAGTACAGGCGG	NM_008855	127
Cacna1e^1,3^	GGAGGTCAGCCCGATGTC	GGGCTCCTCTGGTTGTCC	L29346	420, 399, and 363
Plcd4^2^	TCTCGCGCAATATGCCTTCCAG	ATCTCGGTCAGATGGTGTGCCA	NM_148937	108
Scn8a	CCCGGCAGGAGCCGA	CACTGTTTGGCTTGGGCTTG	NM_001077499.2	235
Plcb1	AGCCAGATGGAAGAGGAGAAG	TCATGGCAACCTTCCGACAA	NM_019677.2	200
Casp8^2^	ATGGCTACGGTGAAGAACTGCG	TAGTTCACGCCAGTCAGGATGC	NM_009812	138

^1^Weiergräber et al. (2005) [58]; Basic Res Cardiol.

^2^Commercially available at http://www.origene.com/.

^3^II-III-loop.

**Table 2:** Fold changes in gene expression of 5XFAD transgenic mice compared to WT controls.

Gene	Fold change
Kcnma1	−1,0643
Cacna2d1	−1,0331
Prkcb	−1,0546
Cacna1e	1,0437
Plcd4	1,6105
Scn8a	−1,0344
Plcb1	−1,1765
Casp8	2,0979

## Abbreviations

A*β*: Amyloid-*β*
A*β*PP: Amyloid-*β* precursor protein
AD: Alzheimer's disease
BACE1: *β*-site APP cleaving enzyme 1
EEG: Electroencephalogram
FAD: Familial Alzheimer's disease
NFTs: Neurofibrillary tangles
PSEN1: Presenilin 1
qPCR: Quantitative real-time PCR
RMS: Root mean square
SEM: Standard error of the mean
WT: Wild-type.
