# Supplementary material for: Altered Theta Oscillations and Aberrant Cortical Excitatory Activity in the 5XFAD Model of Alzheimer's Disease
Source: Neural Plast. 2015 Apr 2;2015:781731. doi: 10.1155/2015/781731 (PMC4398951; doi:10.1155/2015/781731)
Supplement: Supplementary file 1 — Methodological aspects of stereotaxic EEG electrode placement, analysis of theta amplitude, and microarray analysis in WT and 5XFAD mice. [file 781731.f1.pdf]

**Supplementary Information**

**Supplementary Figure 1: Stereotaxic EEG electrode placement. A**) One epidural, differential electrode is placed on the motor cortex (M1), an additional intrahippocampal differential electrode is placed in the CA1 region of the hippocampus. Both pseudoreference electrodes are localized on the cerebellum. **B**) Coronal section (scheme) illustrating the localisation of the deep, intracranial electrode for recording the electrohippocampogram. **C**) Close-up of the deep EEG electrode, the sensing lead of the radiofrequency transmitter and their arrangement on top of the murine skull.

**Supplementary Figure 2: Characteristics of theta amplitude in control and 5XFAD mice.** The theta-alpha band was analyzed using a time-frequency approach. Behavioral artefacts were removed using an algorithm as described above. Amplitude was calculated for both genotypes for the total observation period (**A**) and the light (**B**) and dark (**C**) phase, respectively. Total and phase-specific analysis was also done for non-motor activity (**D-F**) and motor activity (not shown). No difference was observed between both genotypes.

**Supplementary Figure 3: Functional integration of seizure activity and theta oscillations.** Time-frequency analysis in 5XFAD mice revealed changes in theta duration and theta frequency which are not related to motor activity, particularly during the dark phase. Under these conditions, animals are ether at either sleep or at rest. Hippocampal activity during the state of alert immobility is characterized by atropine-sensitive type II theta, which is related to the muscarinic signal transduction pathway. Activation of PLC was shown to enhance $Ca^{2+}$ influx via $Ca_v2.3$ R-type $Ca^{2+}$ channels which can lead to the generation of

epileptiform burst activity via CNG mediated plateau potentials. However, $Ca_v2.3$ R-type $Ca^{2+}$ channels were also shown to be involved in the generation of hippocampal theta oscillations.

**Supplementary Table 1: Representation of interesting candidate genes based on microarray analysis.** The absolute fold change (AFC) between transgenic 5XFAD (TG) mice and WT control littermates.

**Supplementary Table 2: Presentation of candidate genes based on microarray analysis with absolute fold change (AFC) > 2 between transgenic 5XFAD (TG) mice and WT control littermates (WT).** Gene ontology analysis revealed that the strongest transcriptional alterations belong to immune-response and inflammation related genes observed in the context of late-stage cerebral amyloidosis.

Supplementary Figure 1 (Siwek et al., 2015)

A

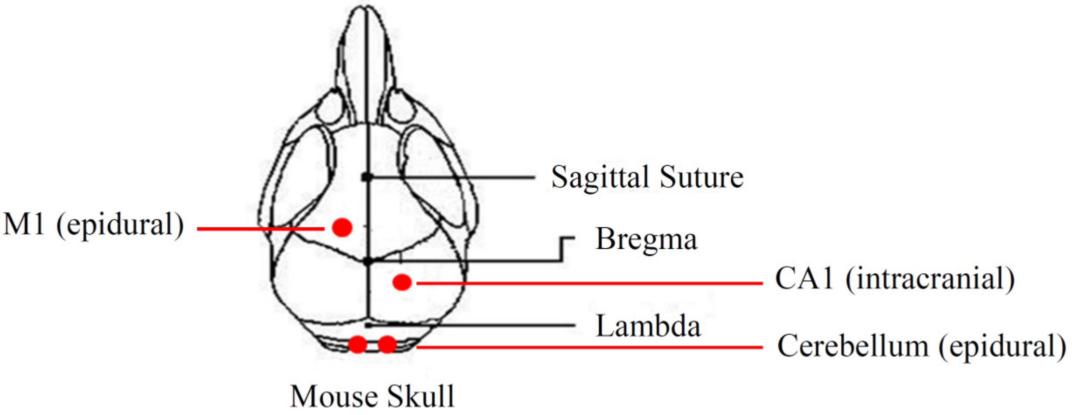


B

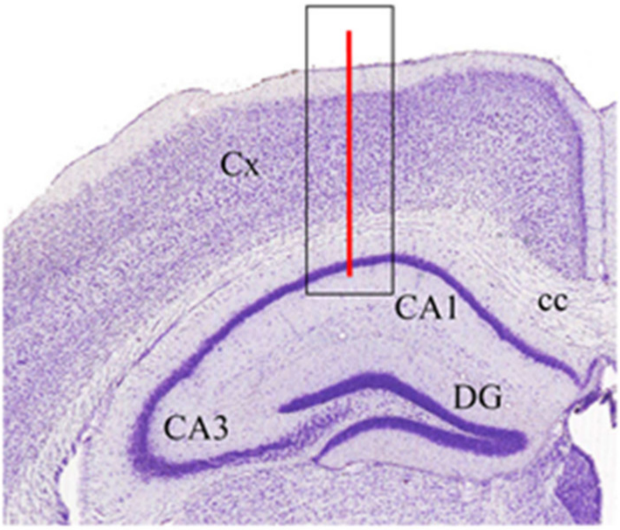


C

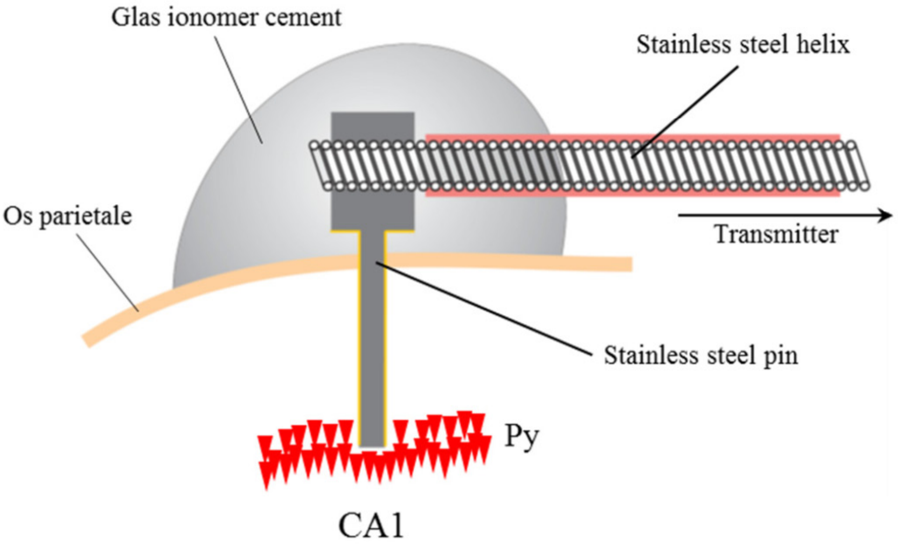

Supplementary Figure 2 (Siwek et al., 2015)

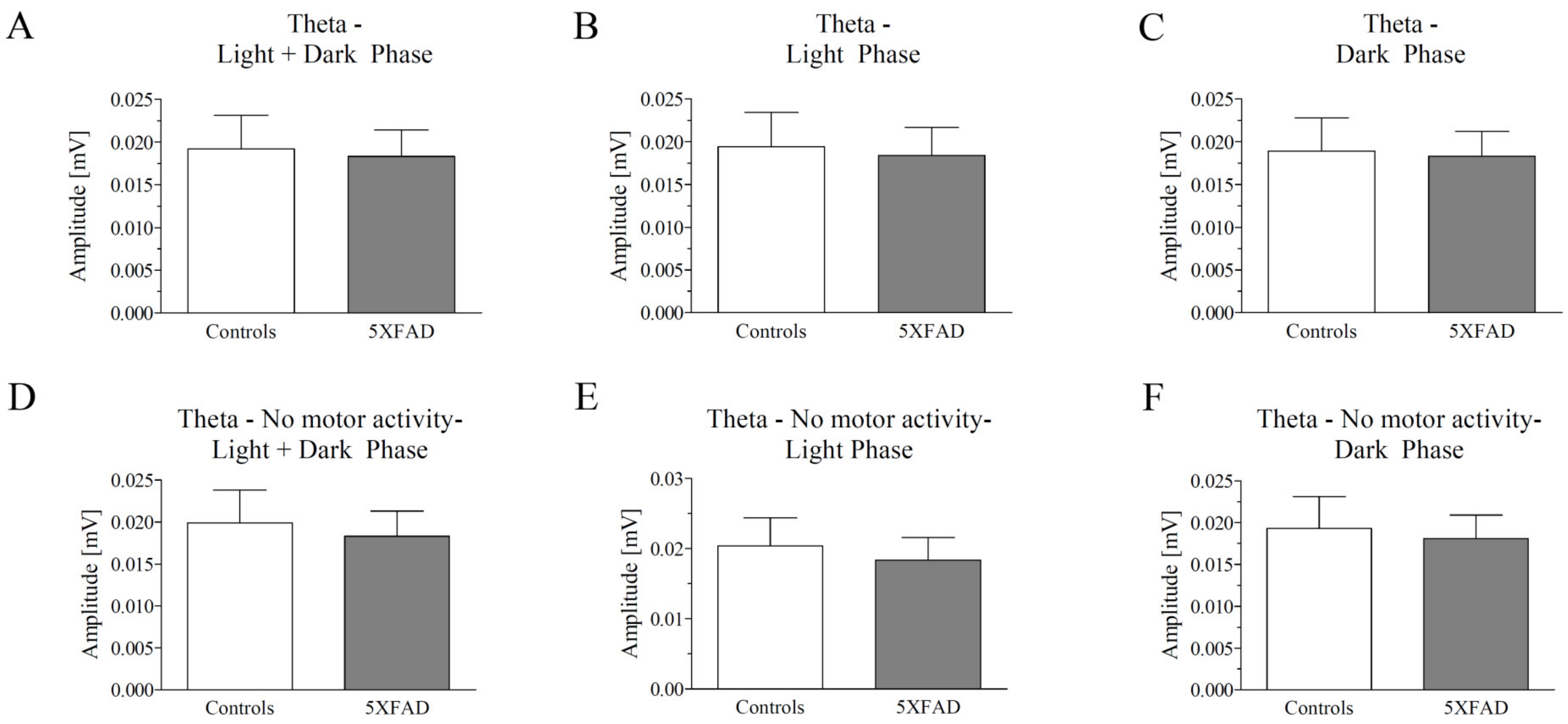

Supplementary Figure 3 (Siwek et al., 2015)

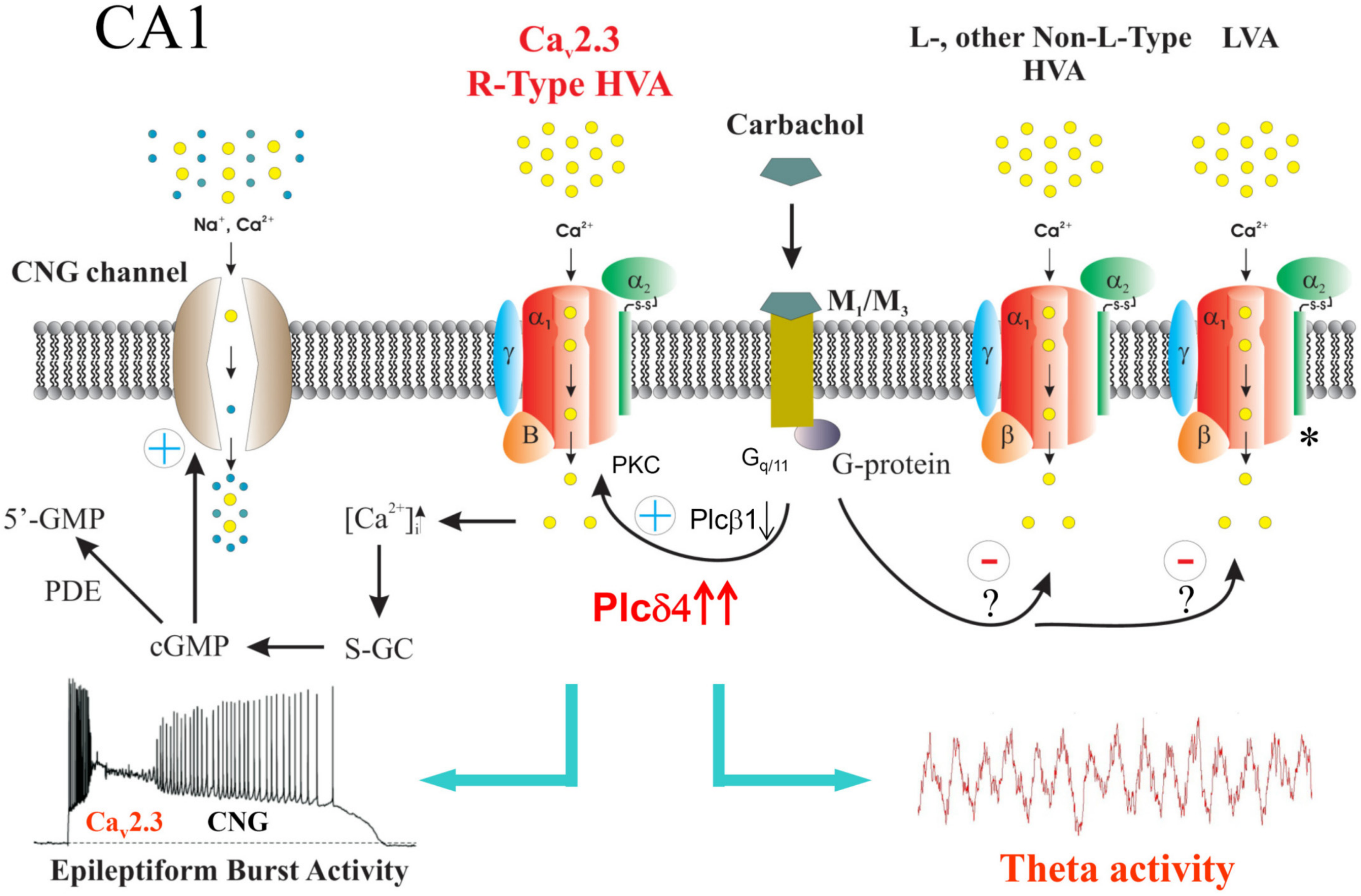

**Supplementary Table 1:**
Representation of interesting candidate genes revealed from microarray analysis. Absolute fold change (AFC) between transgenic Alzheimer's 5XFAD mice (TG) and wild type control littermates (WT).

| **AFC TG vs WT** | Regulation | Candidate Genes |
|---|---|---|
| 1.1079566 | down | Cacna2d1, Calcium Channel, L Type, Alpha |
| 1.1112925 | down | Cacna1e, Cav2.3 calcium channel |
| 1.1162357 | up | Cacng5, calcium channel, voltage-dependent, gamma subunit 5 |
| 1.2483352 | down | Cacng2, calcium channel, voltage-dependent, gamma subunit 2 |
| 1.2776582 | down | Kcna4, potassium voltage-gated channel |
| 1.1231822 | down | Kcnab1, potassium voltage-gated channel |
| 1.0865682 | down | Kcne4, potassium voltage-gated channel, Isk-related family, member 4 |
| 1.3661991 | down | Kcnh5, potassium voltage-gated channel, subfamily H (eag-related), member 5 |
| 1.4835429 | down | Kcnh7, potassium voltage channel, subfamily H, member 7 |
| 1.1978878 | down | Kcnip4, potassium channel interacting protein |
| 1.1670644 | down | Kcnj11, potassium inwardly-rectifying channel, subfamily J, member 11 |
| 1.0783806 | down | Kcnma1, potassium large conductance calcium-activated channel, subfamily M, α1 |
| 1.2284602 | down | Kcnn2, potassium intermediate/small conductance calcium-activated channel, subfamily N, member 2 |
| 1.2311752 | down | Kcnq3, potassium voltage-gated channel, KQT-like subfamily, member 3 |
| 1.1255795 | down | Scn8a, sodium channel, voltage-gated, type VIII, alpha |
| 1.0604281 | up | Ank, progressive ankylosis protein |
| 1.1039068 | down | Ankra2, ankyrin repeat, family A (RFXANK-like), 2 |
| 1.2761725 | up | Ankrd13a, ankyrin repeat domain 13A |
| 1.7455606 | down | Ankrd34c, ankyrin repeat domain 34C |
| 1.1975257 | down | Ankrd43, ankyrin repeat domain-containing protein 43 |
| 1.1477777 | down | Ankzf1, ankyrin repeat and zinc finger domain containing 1 |
| 1.1017654 | up | Aplp2, amyloid beta (A4) precursor-like protein 2 |
| 1.0611709 | down | Calm2, calmodulin 2 |
| 1.4713943 | up | Chrm5, cholinergic receptor, muscarinic 5 |
| 1.158719 | up | Eef1b2, eukaryotic translation elongation factor 1 beta 2 |
| 1.1446922 | down | Gabra4, gamma-aminobutyric acid (GABA) A receptor, subunit alpha 4 |
| 1.2972732 | down | Gabrb2, gamma-aminobutyric acid (GABA) A receptor, subunit beta 2 |
| 1.0849586 | down | Gabrb3, gamma-aminobutyric acid (GABA) A receptor, subunit beta 3 |

| | | |
|---|---|---|
| 1.0767342 | down | Gria3, AMPA-selective glutamate receptor 3 |
| 1.185587 | down | Gria4, AMPA-selective glutamate receptor 4 |
| 1.1791353 | down | Grid1\|Mir346, gluR delta-1 subunit |
| 1.1244333 | down | Grin2a, N-methyl D-aspartate receptor subtype 2A |
| 1.1251612 | down | Grin2b, N-methyl D-aspartate receptor 2B |
| 1.0896682 | down | Kank3, KN motif and ankyrin repeat domains 3 |
| 1.1632725 | down | Negr1, neuronal growth regulator 1 |
| 1.1632118 | down | Pde1b, phosphodiesterase 1B, Ca2+-calmodulin dependent |
| 1.149241 | down | Plcb1, phospholipase C, beta 1 |
| 1.1644183 | up | Plcb3, phospholipase C, beta 3 |
| 1.2514523 | up | Plcd4\|Zfp142, phospholipase C, delta 4, PLC-delta-4 |
| 1.9414148 | up | Plce1, phospholipase C, epsilon |
| 1.2460053 | up | Plcg2, phospholipase C, gamma 2 |
| 1.1797961 | down | Prkar2b, protein kinase, cAMP dependent regulatory, type II beta |
| 1.1495365 | down | Prkcb, protein kinase C, beta |
| 1.1005048 | up | Prkx, protein kinase PKX1 |
| 1.1885214 | up | Rapgef3, Rap1 guanine-nucleotide-exchange factor directly activated by cAMP |
| 1.2769251 | down | Scg2, secretogranin II |
| 1.1976677 | down | Stx1b, syntaxin-1B |
| 1.5812538 | up | Syngr2, synaptogyrin 2 |
| 1.2852507 | down | Trank1, tetratricopeptide repeat and ankyrin repeat containing 1 |
| 1.5507693 | up | Vamp8, vesicle-associated membrane protein 8 |
| 1.5849916 | up | Casp8, Caspase 8 |
| 1.4427319 | up | Cflar, Casp8 and FADD-like apoptosis regulator |

**Supplementary Table 2:**
Presentation of candidate genes revealed from microarray analysis with absolute fold change (AFC) ≥ 2 between transgenic Alzheimer's 5XFAD (TG) mice and wild type control littermates (WT).

| AFC TG vs WT | Regulation | Candidate Genes |
|---|---|---|
| 30,691187 | up | Cst7, cystatin F (leukocystatin);<br>2310001A20Rik, RIKEN cDNA 2310001A20 gene |
| 25,800545 | up | Cst7, cystatin F (leukocystatin);<br>2310001A20Rik, RIKEN cDNA 2310001A20 gene |
| 11,236571 | up | Ccl3, chemokine (C-C motif) ligand 3 |
| 6,5900187 | up | Gpnmb, glycoprotein (transmembrane) nmb |
| 6,0320563 | up | Olfr111, olfactory receptor 111;<br>Olfr110, olfactory receptor 110 |
| 4,5509095 | up | Lyz2, lysozyme 2;<br>Lyz1, lysozyme 1 |
| 4,509695 | up | Tyrobp, TYRO protein tyrosine kinase binding protein |
| 4,4565377 | up | CD68 antigen |
| 4,2304597 | up | Serpina3nm, serine (or cysteine) peptidase inhibitor, clade A, member 3N |
| 4,0053725 | up | Osmr, oncostatin M receptor |
| 3,980827 | up | Trem2, triggering receptor expressed on myeloid cells 2 |
| 3,9256482 | up | Gfap, glial fibrillary acidic protein |
| 3,8248506 | up | C3ar1, complement component 3a receptor 1 |
| 3,6593776 | up | Ly86, lymphocyte antigen 86;<br>Igsf21, immunoglobin superfamily, member 21 |
| 3,6451392 | up | Clec7a, C-type lectin domain family 7, member a |
| 3,6262984 | up | C4b, complement component 4B (Childo blood group);<br>C4a, complement component 4A (Rodgers blood group) |
| 3,5611908 | up | Itgax, integrin alpha X;<br>Mcart1, mitochondrial carrier triple repeat 1;<br>Itgad, integrin, alpha D |
| 3,4145424 | up | Lilrb4, leukocyte immunoglobulin-like receptor, subfamily B, member 4;<br>Gp49a, glycoprotein 49 A |
| 3,4005163 | up | CD180 antigen |
| 3,2523582 | up | CD84 antigen |
| 3,074628 | up | Ctss, cathepsin S |
| 3,0741098 | up | Fcer1g, Fc receptor, IgE, high affinity I, gamma polypeptide |
| 3,0641675 | up | Fcgr3, Fc receptor, IgG, low affinity III |
| 3,0616438 | up | Lgals3bp, lectin, galactoside-binding, soluble, 3 binding protein |
| 3,0302508 | up | C1qc, complement component 1, q subcomponent, C chain |
| 2,9237657 | up | C1qb, complement component 1, q subcomponent, beta polypeptide |
| 2,905447 | up | Laptm5, lysosomal-associated protein transmembrane 5 |
| 2,8482487 | up | C1qa, complement component 1, q subcomponent, alpha polypeptide |
| 2,8451812 | up | Fcgr2b, Fc receptor, IgG, low affinity IIb |

| | | |
|---|---|---|
| 2,8256595 | up | Ctsd, cathepsin D |
| 2,8111982 | up | Plek, pleckstrin |
| 2,8064747 | up | Ctsz, cathepsin Z |
| 2,7769005 | up | Hexb, hexosaminidase B |
| 2,7556384 | up | Tlr7, toll-like receptor 7 |
| 2,615431 | up | Bcl2a1d, B-cell leukemia/lymphoma 2 related protein A1d;<br>Bcl2a1b, B-cell leukemia/lymphoma 2 related protein A1b;<br>Bcl2a1a, B-cell leukemia/lymphoma 2 related protein A1a |
| 2,543658 | up | Slc11a1, solute carrier family 11 (proton-coupled divalent metal ion transporters), member 1 |
| 2,4172409 | up | CD52 antigen |
| 2,4062214 | up | CD9 antigen |
| 2,3877494 | up | Slc14a1, solute carrier family 14 (urea transporter), member 1 |
| 2,385867 | up | CD53 antigen |
| 2,3474405 | up | Cybb, cytochrome b-245, beta polypeptide |
| 2,3225377 | up | Lcn2, lipocalin 2 |
| 2,3032408 | up | Hvcn1, hydrogen voltage-gated channel 1;<br>Tctn1, tectonic family member 1 |
| 2,2871568 | up | Gp49a, glycoprotein 49 A;<br>AB056442, cDNA sequence AB056442;<br>Lilrb4, leukocyte immunoglobulin-like receptor, subfamily B, member 4;<br>D630002G06Rik, RIKEN cDNA D630002G06 gene |
| 2,2849452 | up | Csf3r, colony stimulating factor 3 receptor (granulocyte) |
| 2,2655497 | up | Gm11428, predicted gene 11428 |
| 2,2569838 | up | Irf8, interferon regulatory factor 8 |
| 2,231863 | up | H2-K1, histocompatibility 2, K1K region;<br>Gm7035, predicted gene 7035;<br>H2-Q2, histocompatibility 2, Q region locus 2;<br>LOC100044874, similar to H-2K(d) antigen |
| 2,2273088 | up | Cybrd1, cytochrome b reductase 1 |
| 2,1967485 | up | Tlr13, toll-like receptor 13 |
| 2,1918335 | up | 4632428N05Rik, RIKEN cDNA 4632428N05 gene |
| 2,191425 | up | Cx3cr1, chemokine (C-X3-C) receptor 1 |
| 2,182198 | up | H2-D1, histocompatibility 2, D region locus 1;<br>H2-Q2, histocompatibility 2, Q region locus 2;<br>H2-L, histocompatibility 2, L region;<br>H2-K1, histocompatibility 2, K1, K region |
| 2,1029935 | up | Hpgds, hematopoietic prostaglandin D synthase |
| 2,0661345 | up | Nckap1l, NCK associated protein 1 like |
| 2,040309 | up | Csf1r, colony stimulating factor 1 receptor |
| 2,0336578 | up | Havcr2, hepatitis A virus cellular receptor 2 |
| 2,0043225 | up | Man2b1, mannosidase 2, alpha B1 |
| 2,001403 | up | Trf, transferrin |
